# Shifting the paradigm of music instruction: implications of embodiment stemming from an augmented reality guitar learning system

**DOI:** 10.3389/fpsyg.2014.00471

**Published:** 2014-05-27

**Authors:** Joseph R. Keebler, Travis J. Wiltshire, Dustin C. Smith, Stephen M. Fiore, Jeffrey S. Bedwell

**Affiliations:** ^1^Training Research and Applied Cognitive Engineering Laboratory, Department of Psychology, Wichita State UniversityWichita, KS, USA; ^2^Cognitive Sciences Laboratory, Institute for Simulation and Training, University of Central FloridaOrlando, FL, USA; ^3^Department of Philosophy, University of Central FloridaOrlando, FL, USA; ^4^Psychophysiology of Mental Illness Laboratory, Department of Psychology, University of Central FloridaOrlando, FL, USA

**Keywords:** embodied music cognition, music, augmented reality, training, guitar instruction

## Abstract

Musical instruction often includes materials that can act as a barrier to learning. New technologies using augmented reality may aid in reducing the initial difficulties involved in learning music by lowering these barriers characteristic of traditional instructional materials. Therefore, this set of studies examined a novel augmented reality guitar learning system (i.e., the Fretlight® guitar) in regards to current theories of embodied music cognition. Specifically, we examined the effects of using this system in comparison to a standard instructional material (i.e., diagrams). First, we review major theories related to musical embodiment and specify a niche within this research space we call *embodied music technology for learning*. Following, we explicate two parallel experiments that were conducted to address the learning effects of this system. Experiment 1 examined short-term learning effects within one experimental session, while Experiment 2 examined both short-term and long-term effects across two sessions spaced at a 2-week interval. Analyses demonstrated that, for many of our dependent variables, all participants increased in performance across time. Further, the Fretlight® condition consistently led to significantly better outcomes via interactive effects, including significantly better long term retention for the learned information across a 2 week time interval. These results are discussed in the context of embodied cognition theory as it relates to music. Potential limitations and avenues for future research are described.

## Introduction

Learning a popular-musical instrument, like the guitar, is an experience often accompanied by very informal learning processes. However, outside of the classical repertoire or formal instruction, there simply is no required or specified way to learn an instrument (Green, 2008). Whether formal or informal, learning to play a musical instrument is an embodied process that involves both perception and action (Dourish, 2001; Windsor and de Bézenac, 2012). Specifically, the playing of an instrument creates a rich experience that allows individuals to be engaged both physically and mentally, and thus leads to a stronger interconnection of auditory and visual stimuli with motor responses (Birchfield et al., 2008), sometimes called an *action-reaction cycle* (Leman, 2008). This form of sensorimotor integration, in which motor actions, auditory signals, and visual information become coupled and lead to a strengthening of the connections between the associated regions of the brain (Zatorre et al., 2007; Wan and Schlaug, 2010), is integral to the playing of musical instruments (Maes et al., 2013). Additionally, learning to play an instrument is an embodied process in which knowledge of music and performance on an instrument can arise from the co-occurring perceptions and actions that develop in, and constitute, the learning process itself (Alerby and Ferm, 2005; Matyja, 2010; Krueger, 2014). In contrast to information processing approaches to music cognition, the embodied perspective posits that there is a multi-modal link between perception and action that couple the environment and the brain through the body's sensorimotor system (Clark, 1999; Leman, 2008; Krueger, 2009; Nijs et al., 2009; Matyja, 2010; Davis et al., 2012; Maes et al., 2013). In this manner, the boundaries of musician and instrument become eroded (cf. Nijs et al., 2009; Anderson et al., 2012) into what can be thought of as a *human-musical instrument system*.

The present work describes a set of studies that investigate a novel method for informal guitar learning. To lay the theoretical foundation for our research, we discuss theories of grounded cognition, the ecological perspective, and Leman's (2008) Action-Reaction cycle. These provide a representative discussion of what can be considered an embodied approach to music cognition (cf. Alerby and Ferm, 2005; Barsalou, 2008; Leman, 2008; Krueger, 2009; Matyja, 2010; Davis et al., 2012). We then describe several relevant novel musical learning technologies, with specific focus on the Fretlight® guitar. From this, we elaborate on how this learning system provides instructional information directly onto the musical instrument. We argue that this may help to mitigate initial barriers to learning an instrument by reducing the need for a transformational process between external representation (e.g., tablature) and the instrument itself. Based upon our theoretical foundation, we suggest that this type of system could be considered an embodied technology learning system.

A number of theories are relevant to understanding embodied music cognition. The theory of *grounded cognition*, for one, posits that our brains rely on modal, rather than amodal, systems to represent the world (Barsalou, 2008; Pezzulo et al., 2013). In short, this means that sensory information is not abstracted upon and stored amodally, but rather that such information is intrinsically tied to the perceptual modality that gave rise to it. While this view is seemingly recent in the history of cognitive science, it is in fact an ancient view dating back to the works of the Greek philosopher Epicurus (Barsalou, 2008). Barsalou argued that information processing and computational models of cognition do not adequately link higher-order cognitive processes to the actions enabled by them. Further, many theories of cognition simply ignore the influence and role of the body and its movements (Clark, 1999; Krueger, 2009). Grounded cognition attempts to redress this gap through simulation mechanisms that operate upon modal representations (see Barsalou, 2008 for review of evidence for such mechanisms).

Grounded cognition would likely characterize the process of playing the guitar as an interaction between the modal representations of bodily states associated with holding, playing, and hearing the instrument; the prospective modal simulations required for evaluating the created sounds to those expected; and the preparation of the body to act to produce the upcoming musical sequence. From this viewpoint, the representation of the instrument is not just the information defined by an abstract a modal construct, but instead the integration of multi-modal information dynamically shifting in reaction to the instrument and one's interaction with it. This explains the immediate and emergent processes that occur while playing, but also gives us a theoretical approach that explains how an individual learns, acts, and becomes an expert while using a specific instrument across time (Reybrouck, 2006). In other words, grounded theories give us insight into the coupling of perception, action, and cognition that comprise the *human-musical instrument system* from the first few notes created by a novice up and through the most complex music performed by a master expert. However, to the best of our knowledge, grounded cognition research has not specifically been applied to music instrument learning.

Another important theory that lends insights to embodied music cognition is Gibson's (1977) ecological perspective. The ecological perspective is aligned with grounded theory in that both place a greater emphasis on the role of the body in cognition when compared to traditional information processing approaches; however, they are fundamentally distinct in that the ecological approach does not necessitate the use of representations (Windsor and de Bézenac, 2012; Chemero, 2013). Instead, the ecological perspective emphasizes the mutual relationship between an organism and its environment in terms of the action possibilities available to that organism's perceptual systems—a notion characterized by the concept of *affordances* (Gibson, 1979). Affordances contribute to understanding embodied music cognition through the central idea that each organism has a morphological structure referred to as *effectivities* that constrain that organism's interaction with the environment. These effectivities include an organism's “size, shape, muscular structure, movement capacities, needs and sensitivities that make action in the environment possible” (Windsor and de Bézenac, 2012, p. 104; Shaw and Turvey, 1981).

With regard to musical instruments, Windsor and de Bézenac (2012) note that the morphology of the instrument itself is intentionally constructed so as to fit the effectivities of the human body. This is readily apparent in the design of a guitar in that the neck size and spacing of the frets is typically appropriate for the common human hand size. Specifically, the neck affords grasping, and the frets afford evenly spacing fingers into one fret each. Not only do physical dimensions of an instrument couple with human effectivities, but the types of musical structures available to create are in turn afforded by the instrument. In this way, musical instruments often “come to embody the effectivities of their users and possess inbuilt affordances” (Windsor and de Bézenac, 2012, p. 109). Such affordances, or opportunities for interaction with the instrument, are typically motor-based and have been argued to provide the basis for musical understanding (Menin and Schiavio, 2012). With regard to learning, affordances can play a significant role in competence acquisition and, in some cases, musicians adapt their effectivities to enable new action possibilities with their instrument (e.g., training the hand to perform a technique it initially was unable to accomplish; Windsor and de Bézenac, 2012).

Glenberg et al. (1998, p.651) argue that the perception of affordances is triggered simply by perceiving the relevant objects in the environment, which in turn “clamp” our cognitive system. On this view, perception of an object cues the action opportunities available for interacting with that object and cognitive and neural evidence for what might be considered action representations by grounded cognition researchers has been found (Tucker and Ellis, 2004; Helbig et al., 2006; Bub et al., 2008). Further, affordances have been linked to research on canonical neurons (Garbarini and Adenzato, 2004). The activation of these types of neurons has been shown to provide both visual and motor specificity of object shape and function (Rizzolatti and Fadiga, 1998; Garbarini and Adenzato, 2004). From this, one might argue that the guitar could also elicit a similar response (cf. Menin and Schiavio, 2012). More generally, through this coupling of perception and action, philosophers have argued that the cognitive system becomes extended (e.g., Clark and Chalmers, 1998; Krueger, 2014). In fact, recent work has articulated that musical instruments become cognitive extensions of the player (Magnusson and Magnusson, 2013) or *epistemic tools.* In this sense, musical instruments are a special type of tool associated with a highly advanced symbolic system (i.e., music) that directly affects interaction and use (Glenberg and Robertson, 2000; Magnusson, 2009). Although the aforementioned ideas can contribute to developing a theory of embodied music cognition, research along these lines is still relatively nascent. Therefore, we next describe Leman's (2008) Action-Reaction Cycle and suggest that this provides a useful guide for research aligned with our ideas of embodied music cognition, particularly with regard to music learning.

**Figure 1:**
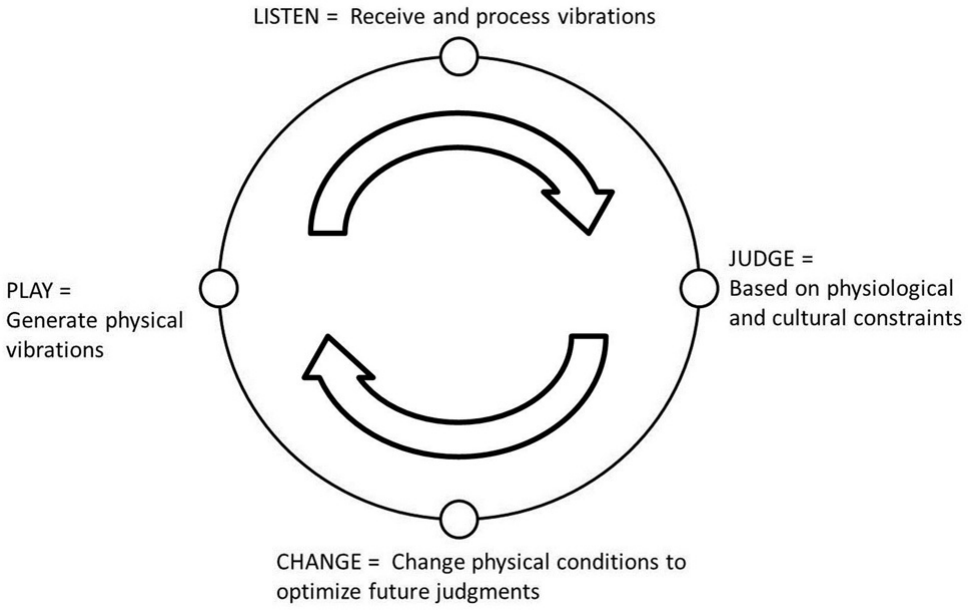
**Adapted from Marc Leman's (2008) Action-Reaction Cycle for embodied music cognition**.

Stated most simply, Leman's (2008) four-step Action-Reaction Cycle (see Figure 1) describes a form of embodied music cognition process that, we suggest, provides an important basis for understanding embodiment in the context of learning to play an instrument. The first step is that an individual must take an action by playing the instrument in some way. Actions create the physical vibrations of the instrument that, in turn, manipulate the air molecules that are perceived as sound by the auditory system. This leads to the second step of the cycle in which the individual listens and processes the sounds they just played. The next step is making a judgment about the quality of that sound as to whether it was the expected sound to be produced. Based upon this judgment, the individual will then make any physical changes in preparation for the next action such that future judgments of the produced sounds will be optimized. In short, Leman's (2008) Action-Reaction Cycle specifies the type of recursive perception-action processes that occur during the embodied engagements that unfold while interacting with an instrument. However, the cycle is limited in the degree to which it articulates the instrument learning process.

**Figure 2:**
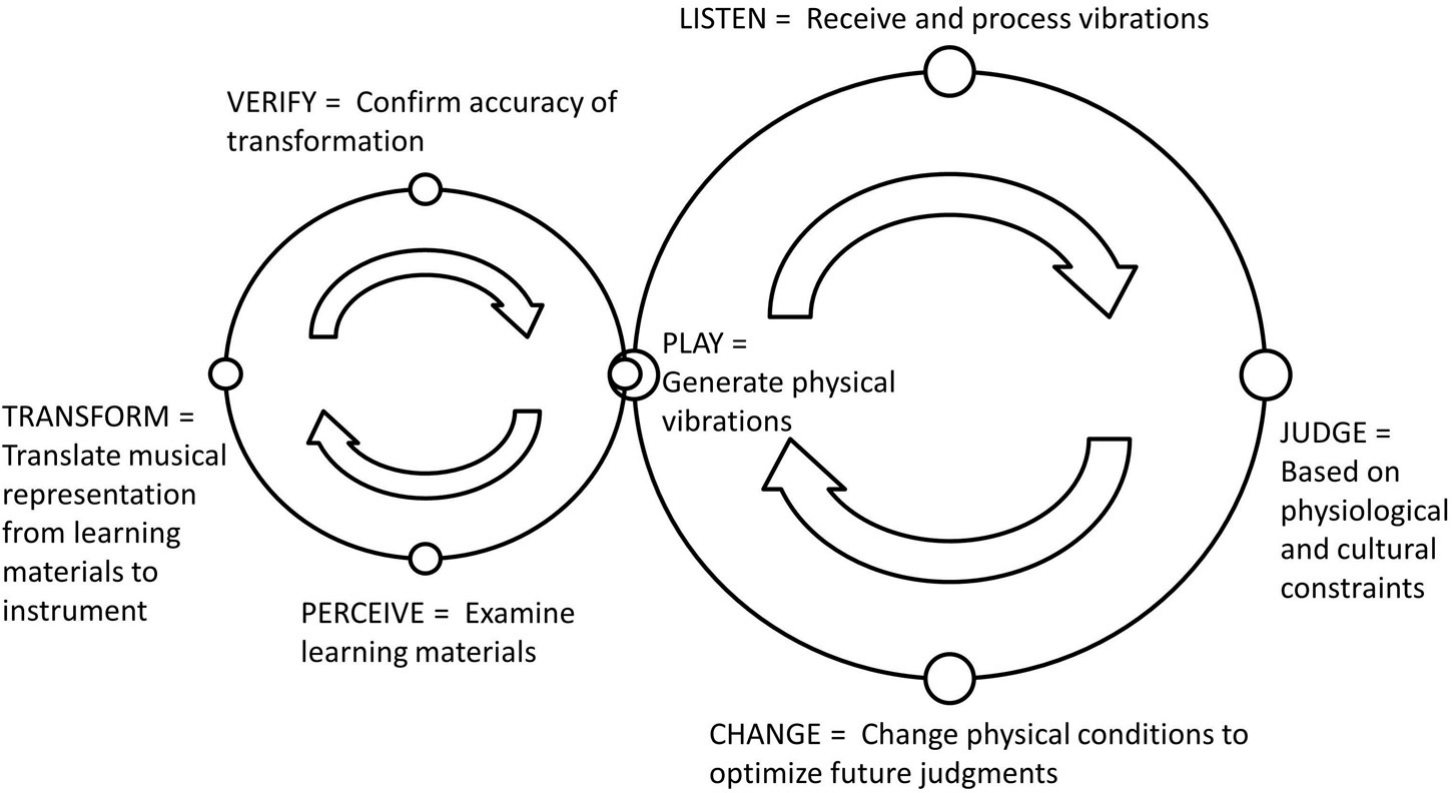
**Updated version of the Action-Reaction Cycle in the context of learning to play a musical instrument and using distributed music learning materials**.

Although music learning processes can be characterized from the embodied perspective, the most common methods for learning an instrument actually run counter to what we consider embodied engagement. This highlights a crucial need for additional steps within Leman's (2008) Action-Reaction Cycle. This barrier takes form in the distributed nature of the materials used for learning to play an instrument (Flor and Holder, 1996). For example, instrument learning materials include sheet music, tablature, chord diagrams, books, audio files, and instructors. The integration of material from numerous external modalities often poses a challenge for the learner, particularly, beginners (e.g., McDermott et al., 2013). Thus, we posit that an additional sub-cycle be added to the Action-Reaction cycle—especially for cases where individuals are learning an instrument through distributed or externalized learning materials such as diagrams, tablature, and musical notation (McDermott et al., 2013). In particular when such externalizations are involved, this sub-cycle characterizes the ways that an individual first *perceives* those external learning materials, how they *transform* them from the external representation to something onto their instrument, and engage in a *verification* of the accuracy of the transformation before actually *playing* a piece of music in accord with the traditional Action-Reaction cycle (see Figure 2). While the experiments detailed herein are not a test of this model *per se*, the model serves an illustrative purpose to scaffold our empirical work with theoretical conceptualizations. To better understand the revised Action-Reaction Cycle, we next discuss novel technological tools used to facilitate guitar learning.

A number of methods and/or technologies have been developed to support music learning by making techniques more accessible to the learner. At a general level, the need to do so is evident in the evolution of musical notation into diagrams or guitar tabs. But, such methods do not resolve the problem that arises from distributed and externalized learning materials described above. More recently, methods have been developed to reduce the need for distributed materials (i.e., musical notation) by providing information directly on or near an instrument. Some of these systems use digital projections (Liarokapis, 2005; Motokawa and Saito, 2006; Shiino et al., 2013) color coded information (Kerdvibulvech and Saito, 2007, 2008), or lights (cf. Keebler et al., 2013) to present the musical information to the player.

One of the major problems with many of these systems is that they are more of a proof of concept and, therefore, focus on technological development instead of their effects on learning. Granted, some have looked at participant ratings of the usability of the augmented reality system as an instrument learning tool (Liarokapis, 2005); but, to the best of our knowledge, no one has evaluated learning itself. Therefore, the current research focuses on an emergent consumer technology that is widely available to the general public, including researchers. We focus on Optek's Fretlight®, which is a novel guitar learning system that shows learners where to place their fingers through light-emitting diodes (LED) embedded just beneath the surface of the guitar's fretboard. This system was designed to lead to more efficient learning and this is explored in the present study. Specifically, we examine if this system leads to faster acquisition of the basics of the guitar (e.g., playing notes and scales).

To better elaborate on the theoretical foundation of embodied music cognition and its relation to augmented music learning systems such as the Fretlight®, an important distinction needs to be made. In terms of learning, researchers have noted that materials and technologies for learning an instrument can emphasize internalized (i.e., use of one's knowledge/skills) and externalized (i.e., available in the environment) modes of learning (Van Nimwegen et al., 2004; McDermott et al., 2013). Learning techniques that rely on internalization come with a greater initial difficultly for the learner, but lead to better long-term retention. However, although externalized learning techniques may be less beneficial in the long term, they provide a lower level of initial difficulty (Van Nimwegen et al., 2004). This is important because, an individual faced with too much initial learning difficulty (e.g., through internalized techniques) may not choose to continue to learn the instrument (McDermott et al., 2013).

With respect to how technologies such as Fretlight® can influence learning, its important to consider the manner in which traditional instructional materials are used. Learners have to first translate instructional materials and music notation into visual-spatial-temporal information. Then, they must translate this into accurate and precisely timed motor movements of the hands (Norton et al., 2005). But, when instructional materials become embodied and embedded within the instrument (cf. Fishkin, 2004), the system should, in theory, allow for a reduction in this translation process. This, in turn, would provide a stronger coupling between multi-modal perceptions and motor responses involved in learning the guitar. In other, words, the augmented reality learning system provided by the Fretlight® utilizes direct information (i.e., a lighted fretboard) to clarify where the body needs to interact in space and time. We suggest that this process aligns well with what we described above as a form of learning with embodied music cognition. It could even be argued that the Fretlight® guitar comes with a richer repertoire of affordances (i.e., opportunities to play to the instrument) simply not available in traditional guitars. In other words, when the guitar indicates what to play, the interaction possibilities of the player should increase.

We want to note, though, that this is a distinct characterization of embodied music cognition in that the material required for learning the instrument actually becomes embodied and embedded in the instrument itself. Traditionally, embodied music cognition is conceptualized as “the ways in which our interaction with music is grounded in movement, feeling and expression” (Matyja, 2010. p. 5) and even extends to, and has implications for, social and cultural interactions (e.g., Phillips-Silver and Keller, 2012; Windsor and de Bézenac, 2012). Our view complements these views to suggest that Fretlight® is an embodied technology that allows for the “offloading” of cognition on to the instrument. This is in line with conceptions of extended cognition (Clark and Chalmers, 1998) in that this diminishes the need to engage in mental transformations such as those required to translate external musical notation onto an instrument.

**Figure 3:**
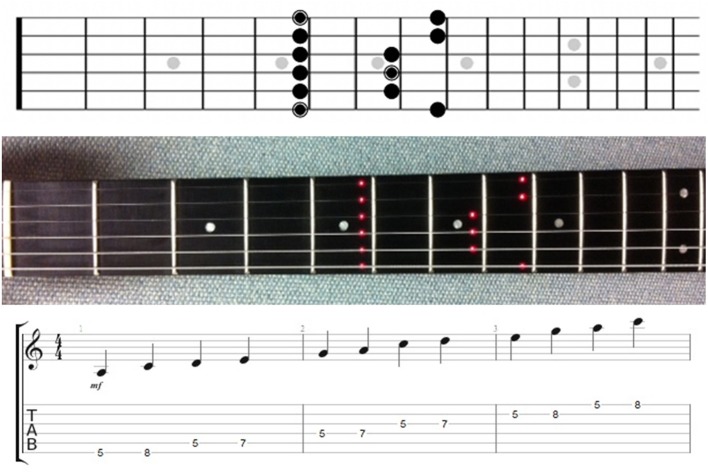
**A minor pentatonic scale diagram (top) and Fretlight® guitar (middle) used in Experiments 1 and 2**. Circled notes on the diagram are the roots (i.e., A in three different octaves), but this was not mentioned to participants. For illustration purposes only, we have also included a musical notation staff and guitar tab (bottom) showing the scale and the notes participants were required to play: A-C-D-E-G-A-C-D-E-G-A-C (The staff and guitar tab were not included in this study).

When playing a Fretlight® guitar a player reaches to put their hand around the neck as with any guitar, but further, they also place their fingers in a form that is natural to playing the scalar/chordal information being presented by the guitar neck (see Figure 3). This may ultimately aid in making the instrument more “transparent” (Nijs et al., 2009). In this context, transparency is viewed as the implicit representation of the instruments form and functional characteristics to the musician. By embedding the learning content on the instrument, it may be that Fretlight® fosters these developmental processes. Specifically, we expect that having the musical information presented directly on the guitar will allow a player to remain in the embodied engagement characterized by the original Action-Reaction cycle (see Figure 1). This is in contrast to traditional instructional materials, which are external to the learner and the instrument, and which necessitate a greater degree of cycling through the revised component of the Action-Reaction proposed in Figure 2.

Given the above, we suggest that such technology can help us re-conceptualize the way musicians interact with instructional content during the learning process (e.g., McDermott et al., 2013). While traditional external learning materials tend to necessitate cognitive transformations (e.g., Norton et al., 2005), systems such as the Fretlight® guitar, while still an external learning material, may reduce the need for the transformation processes detailed in the revised Action-Reaction cycle (see Figure 2). We argue that this will be the case because the musical information is provided on the actual instrument. Therefore, what makes this approach unique is that it adds information directly to the instrument-hand interface. When this system, that affords the instructional information, is compared to learning from external sources, such as sheet music or diagrams, we expect to see learning gains. In other words, we argue that the difficulty associated with both knowing how to read the externalized sources, as well as the challenge in processing and translating that information into motor control movements, will be reduced or removed due to the direct presence of the instructional information on the instrument. This may allow for the learners attention to focus solely on the instrument, as opposed to distributed across various locations in the environment.

In short, we have attempted to highlight some of the theoretical issues surrounding embodied music cognition and specifically cast the Fretlight® guitar as an embodied technology learning system (cf. Fishkin, 2004). We have specifically noted that this type of learning system may prove useful in lowering the learning barrier presented by distributed and externalized music learning materials. Further, we have posited a revision to Leman's (2008) Action-Reaction Cycle to theoretically scaffold our empirical work. In order to investigate the effects of the Fretlight® guitar as a learning system, a program of research was developed between two large U.S. universities (preliminary results can be found in Keebler et al., 2013). This manuscript reviews two initial experiments that examined the effects of using the Fretlight® guitar to learn a basic musical scale when compared to a more standard method of learning (i.e., guitar neck diagrams). Each experiment is discussed with detailed objectives, followed by an individual method, results, and discussion. We conclude with a general discussion that relates the findings from both experiments and offer recommendations for future research in this area.

## Experiment 1

Experiment 1 was conducted in order to examine the differences in performance when learning the A minor pentatonic scale with the Fretlight® guitar as opposed to learning the scale with a diagram. The a priori hypotheses for this experiment were as follows:

“H1: There will be interaction effects between (1) condition and (2) the number of training trials on: scale note quality, errors, and inconsistency between notes. The pattern of improvement over time intervals will differ between the two learning conditions such that rate of improvement of time will be accelerated for the Fretlight® training condition when compared to the diagram condition.”

“H2: There will be interaction effects between (1) condition and (2) the number of testing trials on: scale note quality, errors, and inconsistency between notes. The pattern of improvement over time intervals will differ between the two learning conditions such that rate of improvement of time will be accelerated for the Fretlight® training condition when compared to the diagram condition.”

Given the above theorizing, when considering embodied engagement and the rightmost loop of the revised Action-Reaction cycle (see Figure 2), there may also be effects in the time-dependent measures comprising the construct of fluency (detailed in the Method Section). That is, participants in the Fretlight® condition may demonstrate improved learning times. However, these predictions are more speculative and not formally hypothesized. As such, further elaboration will be presented in the discussion section.

### Methods

#### Participants

55 undergraduate students (30 female, 25 male) from a large southeastern university voluntarily participated in this study in exchange for class credit. Age data were not collected in this experiment; however, participants were required to be at least 18 years of age. To participate in this study individuals must also have been right-handed and must have stated that they never had any formal or informal stringed instrument training. Due to the inability to adhere to the requirements of the experiment, the data from one participant was excluded from further analyses.

#### Materials

A Black FG-521 Traditional Electric Guitar was used for both conditions in the experiment. The program Fretlight studio v5.02 was used to control the display of the LED lights for participants using the Fretlight® learning method. For those in the diagram condition, a paper diagram was provided to participants (see Figure 3). A Logitech HD Webcam C525 Portable HD 720 p was used to record both the video and audio of each participant's performance during the training and test trials. The program Audacity was used to precisely capture time-related dependent variables regarding performance from the audio files (see DVs subsection below).

#### Design

Experiment 1 utilized a mixed design in which participants were randomly assigned to one of two levels of the independent variable (IV). Our between subject IV for this study was the specific *learning method* used to learn the A minor pentatonic scale: Fretlight® learning method or a diagram learning method. In the Fretlight® learning method, LED lights illuminated the scale so that participants could see the proper location of notes embedded within the fret board. Conversely, the scale diagram method consisted of a paper diagram with the notes of the scale depicted on the fret board (Figure 3). Notably, all participants used the same guitar with the difference being that in the diagram condition, the Fretlights® were not on and the instrument thus appeared to be a regular guitar. Participants were given 30 training trials to learn the scale and were then required to complete 10 test trials. Our within subject IV was *time practicing the scale*, which represented the progression of training or test trials. This allowed us to examine learning performance over time.

The following dependent variables (DVs) were used to assess participants' training and test performance: *scale note quality, errors*, and *inconsistency between notes.* In order to determine training and test performance, a performance rating scale was developed to evaluate the quality of each of the 12 notes of the A minor scale and the types of errors for individual testing and training trials (see **Supplementary Material**). Specifically, the *scale note quality* examined accuracy and precision as a function of individual's scores on the rating scale. Performance was determined by assessing each note played by participants and assigning a quality value of 0–4 (e.g., 0 being no note played, 4 being a very well played note; see Performance Rating Section for details). Then the total number of points earned were divided by the total number of possible points for the scale to create a percentage representing scale note quality. *Errors* accounted for instances in which participants played a *wrong note* that was not included within the scale or played an *extra note* beyond the 12 notes required for the scale including repeated notes (See **Supplementary Material** for operationalization of these two types of errors). *Fluency* was determined by a time-dependent measure of *inconsistency between notes* and *total scale time*. To derive these time dependent measures, the total time it took participants to play one note before playing the next note was time-stamped in seconds by our coders for each note in the scale for a given trial. The total time to complete each trial was also time-stamped by the coders. *Inconsistency between notes* was determined by calculating the degree of variance for the time spent on each note before starting the next note within the scale. *Total scale time* was simply the total time in seconds beginning when the participant played their first note till the time the last note in the scale rung out.

#### Procedure

When the experiment first began, all participants were introduced to the guitar and its basics using a PowerPoint presentation and tutorial videos. The videos familiarized participants with how to hold the guitar, how to place the left hand fingers on the fret board, and how to use the right hand to pick the strings. Upon completion of this pre-training familiarization, participants were then randomly assigned to one of the two groups (e.g., Fretlight® or a comparable diagram). Participants then learned the A minor pentatonic scale over 30 training trials. Participants were asked to try their best to play through the whole scale. They were told that if they “mess up” or “miss a note” they should continue to play the next note in the scale. After participants completed the 30 training trials, the learning materials were removed, and each participant was tested across 10 trials. The experiment lasted no longer than 1.5 h.

#### Performance rating

Two individuals familiar with playing the guitar, but naïve to the experimental hypotheses, were trained to apply the rating scale to assess the quality of participant performance for a subset of the training and test trials. In particular, the video and audio files from Training Trials 1, 5, 10, 15, 20, 25, and 30 as well as Test Trials 1, 5, and 10 were rated. This subset was selected given the time intensity involved in scoring performance from video data (cf. Louwerse et al., 2012). Inter-rater reliability was assessed using intra-class correlation coefficients (ICC). Specifically, ICC was deemed the most appropriate metric of inter-rater reliability given the ordinal nature of the scale quality and error variables and the resulting need to account for lower to higher differences in the magnitude of the ratings (Hallgren, 2012). Four individuals familiar with playing guitar but blind to the experiment's hypotheses were trained to apply the rating scale to assess the quality of participant performance. Across all the raters, inter-rater reliability was *ICC* = 0.78. As such, inter-rater reliability can be characterized at an excellent level beyond that due to chance (see Cicchetti, 1994).

### Experiment 1 training performance results

A 2 × 7 mixed-model ANOVA was conducted to examine the effects of the between subjects variable (learning method: Fretlight® or diagram) and the within subjects variable (time practicing the scale: Training Trials 1, 5, 10, 15, 20, 25, and 30) on each of the following DVs: scale note quality, wrong notes, extra notes, inconsistency between notes, and total scale time. Due to violation of Mauchly's assumption of sphericity on each of the subsequent analyses and the fact that the value of ε < 0.75, Greenhouse-Geisser corrections were used to adjust the degrees of freedom (see Girden, 1992). Further, in the cases where significant interactions were found, tests of simple effects were computed with Bonferroni corrections to mitigate the chance of family-wise errors across multiple comparisons associated with these types of tests.

#### Scale note quality

Results showed a main effect of training time, *F*_(4.114,47)_ = 7.39, *p* < 0.001, η^2^_*p*_ = 0.124, such that the mean scale quality score for Training Trial 1 (*M* = 65%) was significantly lower than all the other training trials (*M*_range_ = 71–74%), all *p* < 0.05. However, there was not a main effect for learning method on scale note quality, *F*_(1, 47)_ = 0.48, *p* = 0.483, η^2^_*p*_ = 0.009.

**Figure 4:**
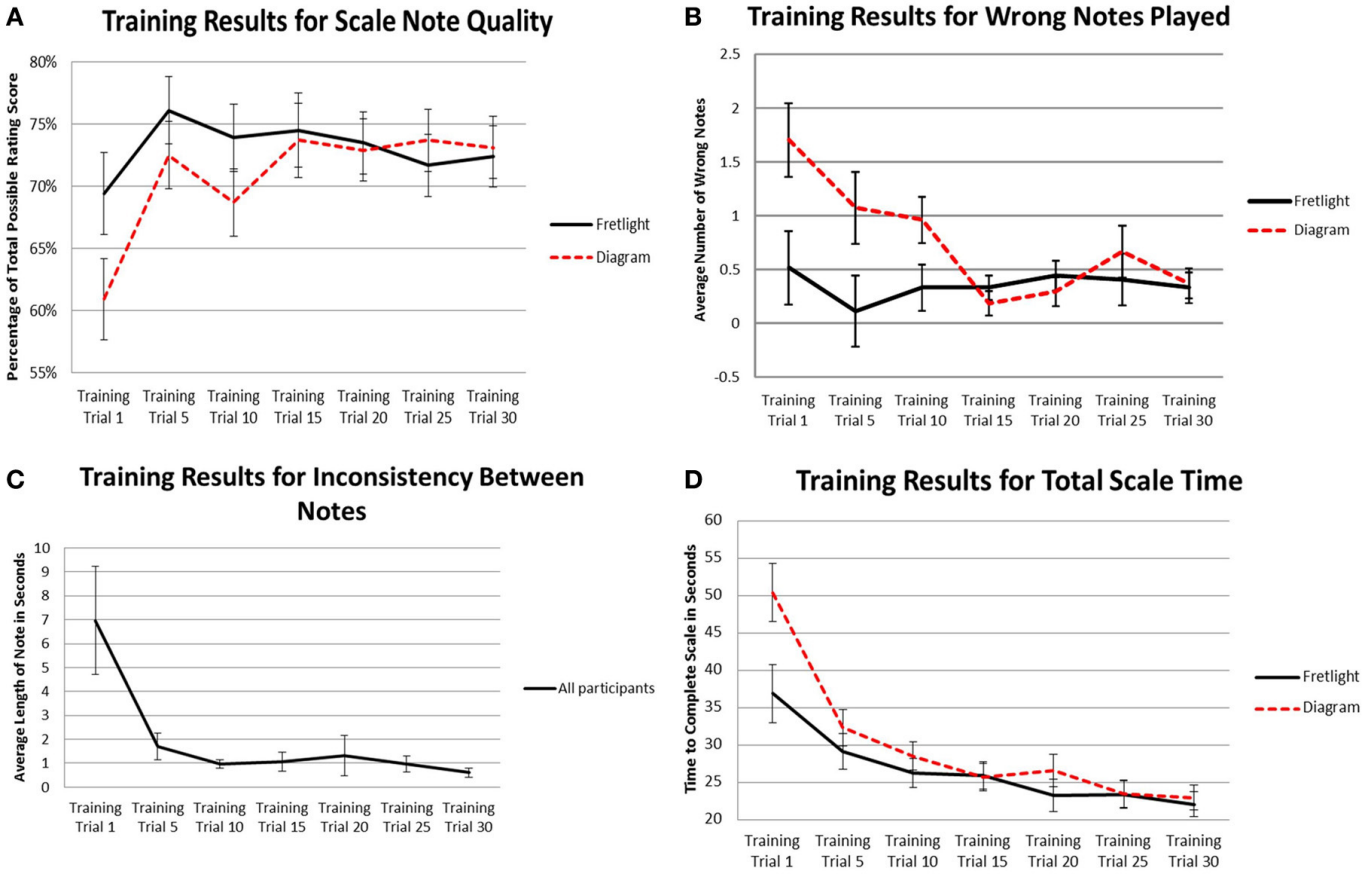
**(A)** Interaction effect of time practicing and learning method on scale note quality; **(B)** Interaction effect of time practicing and learning method on wrong notes played; **(C)** Main effect of time practicing on variation time between notes; **(D)** Interaction effect of time practicing and learning method on total time to complete scale.

There was a significant interaction between time practicing and learning method on scale note quality, *F*_(4.114,47)_ = 2.57, *p* = 0.038, η^2^_*p*_ = 0.047. Results of the pairwise comparisons showed that, when comparing quality of scale notes in the Fretlight® condition to the diagram condition across training trials, scale note quality during the first test trial was non-significantly lower in the diagram condition (*M* = 60.9%) than the Fretlight® condition, (*M* = 69.4%), *p* = 0.075. As shown in Figure 4A, the quality of notes converged between the two conditions throughout the remaining training trials suggesting that group differences disappeared as participants continued to play the scale.

Further, when comparing within a given learning method condition, results of pairwise comparisons showed that scale note quality remained relatively constant for the Fretlight® condition across the training and test trials, *p* > 0.05. Conversely, in the diagram condition, scale note quality was significantly lower during the first training trial (*M* = 60.9%) when compared to all other trials (*M*_range_ = 72.9–73.7%), *p* < 0.05, except for Training Trial 10 (*M* = 68.7%), *p* = 0.066. Thus, participants who were trained to play the scale with the Fretlight® maintained a consistent scale note quality throughout the training trials, while participants in the diagram condition exhibited poorer note quality during the first training trial as compared to the remaining trials.

#### Wrong notes

Results demonstrated a main effect for time practicing the scale, *F*_(3.507,47)_ = 3.99, *p* = 0.006, η^2^_*p*_ = 0.071, such that participants total number of wrong notes decreased as they practiced the scale. The main effect for learning method was not significant, *F*_(1, 47)_ = 3.86, *p* = 0.055, η^2^_*p*_ = 0.069.

A significant interaction between training time and learning method was found on the number of wrong notes played, *F*_(3.507,47)_ = 3.60, *p* = 0.01, η^2^_*p*_ = 0.065. Results of pairwise comparisons showed that, when comparing the number of wrong notes played in the Fretlight® condition to the diagram condition across training trials, the quantity of wrong notes was significantly lower in the Fretlight® condition for Trials 1 (*M* = 0.52, *SE* = 0.34), 5 (*M* = 0.11, *SE* = 0.33), and 10 (*M* = 0.33, *SE* = 0.21) than the same trials in diagram condition, (*M* = 1.70, *SE* = 0.34; *M* = 1.07, *SE* = 0.33; and *M* = 0.96, *SE* = 0.21, respectively), all *p* < 0.05 (see Figure 4B).

Further, when comparing within a given learning method condition, results of pairwise comparisons showed that the number of wrong notes played remained relatively constant for the Fretlight® condition across the training trials, all *p* > 0.05. Conversely, in the diagram condition, the number of wrong notes played decreased significantly when comparing the number of wrong notes played during Training Trial 1 (*M* = 1.70) to Training Trial 15 (*M* = 0.19), Training Trial 20 (*M* = 0.30), and Training Trial 30 (*M* = 0.37), *p* < 0.05. This suggests that in the diagram condition, the number of wrong notes played decreased as the participants practiced the scale. However, participants in the Fretlight® condition played less wrong notes than participants in the diagram condition during earlier training trials but performance levels converged at the end.

#### Extra notes

Results did not show a main effect for training time, *F*_(6, 47)_ = 0.93, *p* = 0.483, η^2^_*p*_ = 0.019, or learning method, *F*_(6, 47)_ = 3.08, *p* = 0.085, η^2^_*p*_ = 0.056. In addition, the results did not show a significant interaction between training time and learning method on total extra notes played, *F*_(6, 47)_ = 0.93, *p* = 0.481, η^2^_*p*_ = 0.021. This means that there were no differences between condition or over time in the number of extra notes played.

#### Inconsistency between notes

Results showed a main effect for training time, *F*_(1.46,47)_ = 5.90, *p* = 0.009, η^2^_*p*_ = 0.102 (see Figure 4C). Therefore, participants played the scale at an increasingly consistent pace across training time. However, there was no main effect for learning method, *F*_(1, 47)_ = 2.19, *p* = 0.145, η^2^_*p*_ = 0.040, and no interaction effect between time practicing and learning method on inconsistency between notes, *F*_(1.46,47)_ = 2.49, *p* = 0.105, η^2^_*p*_ = 0.046.

#### Total scale time

There was a main effect for time practicing the scale, *F*_(2.40,47)_ = 56.41, *p* < 0.001, η^2^_*p*_ = 0.52. Pairwise comparisons showed that the total time to complete the scale significantly decreased from Training Trial 1 (*M* = 43.68) and Training Trial 5 (*M* = 30.76) to all other training and test trials (*M*_range_ = 22.05–27.41), *p* < 0.05. However, there was no main effect for learning method, *F*_(1, 47)_ = 1.39, *p* = 0.244, η^2^_*p*_ = 0.026. That is, the total time to complete the scale did not differ across the two training groups.

Last, the results showed a significant interaction between the number of training trials completed and learning method on total scale time, *F*_(2.40,47)_ = 5.91, *p* = 0.002, η^2^_*p*_ = 0.102 (see Figure 4D). Results of the pairwise comparisons showed that, when comparing total time to complete the scale in the Fretlight® condition to the diagram condition across training trials, total time to complete the scale was significantly lower in the Fretlight® condition during Training Trial 1 (*M* = 36.93) when compared to the diagram condition, (*M* = 50.44), *p* = 0.018. Further, when comparing within a given learning method condition, results of pairwise comparisons showed total time to complete the scale decreased significantly from Training Trial 1 in the Fretlight® condition (*M* = 36.93) to all other training trials (*M*_range_ = 22.08–26.27), *p* < 0.05, except Training Trial 5 (*M* = 29.20). Similarly, in the diagram condition, total time to complete the scale decreased significantly from Training Trial 1 (*M* = 50.44) to all other training trials (*M*_range_ = 21.73–32.33), *p* < 0.001. This means that participants played the scale faster as they progressed through the training trials. The groups differed at Training Trial 1 meaning that participants in the Fretlight® condition initially play faster than participants in the diagram condition but playing time converges at the end.

### Experiment 1 test performance results

For each of the subsequent analyses, a 2 × 3 mixed-model ANOVA was conducted to examine the effects of the between subjects variable (learning method Fretlight® or diagram) and the within subjects variable (Time practicing the scale in terms of the number of trials: Test Trial 1, Test Trial 5, and Test Trial 10) on one of the following DVs: Scale note quality, wrong notes, extra notes, inconsistency between notes, and total scale time.

#### Scale note quality

**Figure 5:**
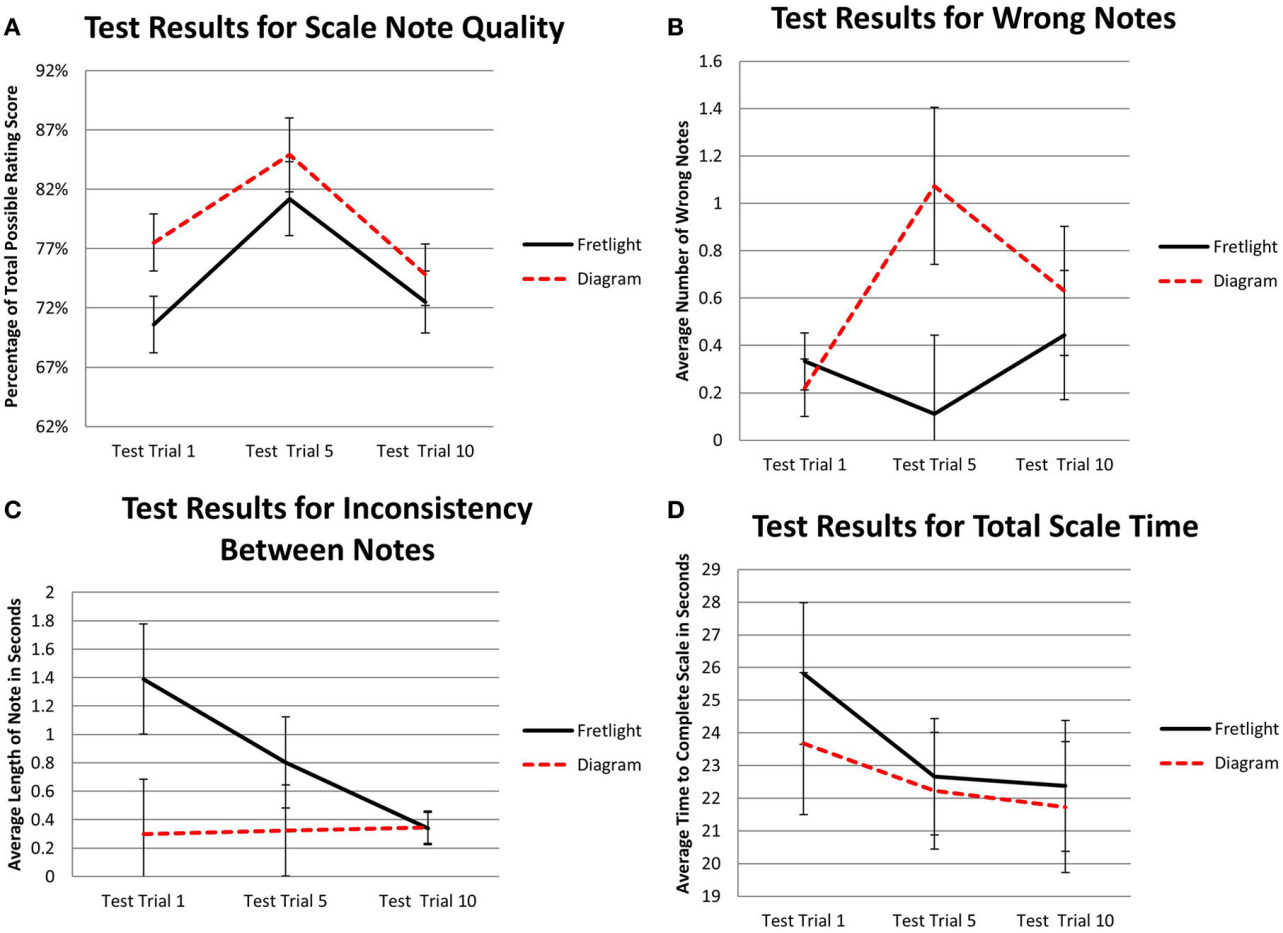
**(A)** Test results for scale note quality; **(B)** Test results for wrong notes; **(C)** Test results for inconsistency between notes; **(D)** Test results total scale time.

Results showed a main effect for time practicing the scale, *F*_(2, 51)_ = 12.72, *p* < 0.001, η^2^_*p*_ = 0.333, such that overall, scale note quality during Test Trial 5 (*M* = 83%) was significantly higher than Test Trial 1 (*M* = 74%) and Test Trial 10 (*M* = 73.7%), *p* < 0.05. However, there was not a main effect for learning method, *F*_(1, 52)_ = 1.80, *p* = 0.186, η^2^_*p*_ = 0.033, or an interaction between time practicing the scale and learning method on scale note quality, *F*_(2, 51)_ = 1.47, *p* = 0.24, η^2^_*p*_ = 0.055 (see Figure 5A). Therefore, it is reasonable to infer that scale note quality did not differ across test trials or groups.

However, given the disparity in performance between the two conditions during the first test trial, as indicated by the none-overlapping error terms shown in Figure 5A, pairwise comparisons were computed to determine if there were statistically significant differences. Results showed that, when comparing quality of scale notes in the Fretlight® condition to the diagram condition in Test Trial 1, scale note quality was significantly lower in the Fretlight® condition (*M* = 70.6%) than the diagram condition, (*M* = 77.5%), *p* = 0.033. However, this difference was no longer evident in Test Trial 5 or Test Trial 10, *p* > 0.05.

#### Wrong notes

Results did not show a main effect for time practicing the scale *F*_(2, 51)_ = 1.51, *p* = 0.23, η^2^_*p*_ = 0.056, or for learning method, *F*_(1, 52)_ = 1.26, *p* = 0.267, η^2^_*p*_ = 0.024. However, there was a significant interaction between time practicing the scale and learning method on the total number of wrong notes played, *F*_(2, 51)_ = 4.22, *p* = 0.02, η^2^_*p*_ = 0.056 (see Figure 5B).

Tests of simple effects were computed to better understand the interaction. Pairwise comparisons suggest that the interaction was driven by a significant difference in the number of wrong notes played during Test Trial 5 with the diagram condition playing more wrong notes (*M* = 1.07) than the Fretlight® condition (*M* = 0.111), *p* < 0.05. There was also a significant increase in wrong notes played in the diagram condition between Test Trial 1 (*M* = 0.222) and Test Trial 5 (*M* = 1.07), *p* < 0.05. Therefore, participants in the diagram condition played more wrong notes than participants in the Fretlight® condition as they progressed through the test trials.

#### Extra notes

Results showed a main effect for time practicing the scale, *F*_(2, 51)_ = 4.59, *p* = 0.015, η^2^_*p*_ = 0.153, such that overall, there was a significant decrease in the total extra notes played between Test Trial 5 (*M* = 0.741) and Test Trial 10 (*M* = 0.315), *p* < 0.05. However, the results did not show a main effect for learning method, *F*_(1, 52)_ = 0.946, *p* = 0.335, η^2^_*p*_ = 0.018, or a significant interaction between time playing and learning method on scale note quality, *F*_(2, 51)_ = 1.49, *p* = 0.236, η^2^_*p*_ = 0.055. Therefore, the time it took participant to play every note in the scale did not vary by groups during test trials.

#### Inconsistency between notes

Results did not show a main effect for time practicing the scale, *F*_(2, 51)_ = 1.65, *p* = 0.201, η^2^_*p*_ = 0.031, or for learning method, *F*_(1, 52)_ = 3.82, *p* = 0.056, η^2^_*p*_ = 0.068. In addition, the results did not show a significant interaction between time practicing the scale and learning method on scale note quality, *F*_(2, 51)_ = 1.99, *p* = 0.147, η^2^_*p*_ = 0.073 (see Figure 5C). As such, the time between each note played did not vary by group as they played through the test trials.

Given the disparity in performance between the two conditions, as indicated by the non-overlapping error bars in Figure 4C for Test Trial 1, pairwise comparisons were computed to further compare the conditions. Results showed that, when comparing the Fretlight® condition to the diagram condition in Test Trial 1, variation time between notes was non-significantly higher in the Fretlight® condition (*M* = 1.39) than the diagram condition, (*M* = 0.297), *p* = 0.052. However, the variation in note length decreased significantly in the Fretlight® condition between Test Trial 1 (*M* = 1.39) and Test Trial 10, (*M* = 0.338), *p* < 0.05.

#### Total scale time

Results showed a main effect for time practicing the scale, *F*_(2, 51)_ = 6.30, *p* = 0.004, η^2^_*p*_ = 0.198, such that overall, the total time to complete the scale during Test Trial 1 (*M* = 24.75) significantly decreased when compared to Test Trial 5 (*M* = 22.44) and Test Trial 10 (*M* = 22.05), *p* < 0.05. However, there was no main effect for learning method, *F*_(1, 52)_ = 0.16, *p* = 0.693, η^2^_*p*_ = 0.003, and no interaction between trial practicing the scale and learning method on total time to complete the scale, *F*_(2, 51)_ = 0.64, *p* = 0.53, η^2^_*p*_ = 0.025 (see Figure 5D). Therefore, the total time to play through the scale for a given test trial did not vary across conditions.

### Experiment 1 discussion

This study demonstrated support for most of our hypotheses during the training phase of the experiment. Table 1 provides an overview of the significant findings. In summary, support for H1 was evidenced by the interaction effects found between both condition and time practicing the scale on almost all of the DVs. Specifically, H1 was supported by the finding that both conditions had increases in performance over the course of the training, as well as by the finding that scale note quality was greater during the first training trial for the Fretlight® condition. Support was also shown for H1 by the finding that there were fewer errors in the Fretlight® condition during Training Trials 1, 5, and 10. Lastly, H1 was further supported by the finding that there was less total scale time during Trial 1 for the Fretlight® condition when compared to the diagram condition.

**Table 1:** **Summary of Experiment 1 Results**.

**DV**	**Effect**
Training scale notes quality	Training trials^*^
	Learning method
	Training trials X learning method^*^^FL^
Testing scale notes quality	Test trials^*^
	Learning method
	Test trials X learning method
Training wrong notes	Training trials^*^
	Learning method
	Training trials X learning method^*^^FL^
Testing wrong notes	Test trials^*^
	Learning method
	Test trials X learning method^*^^FL^
Training extra notes	Training trials
	Learning method
	Training trials X learning method
Testing extra notes	Test trials^*^
	Learning method
	Test trials X learning method
Training inconsistency between notes	Training trials^*^
	Learning method
	Training trials X learning method
Testing inconsistency between notes	Test trials
	Learning method
	Test trials X learning method
Training total scale time	Training trials^*^
	Learning method
	Training trials X learning method^*^^Fl^
Testing total scale time	Test trials^*^
	Learning method
	Test trials X learning method

* *p < 0.05*,

Interaction effects are qualified with superscript to indicate which condition showed an advantage where ^FL^, Fretlight®; ^D^, Diagram.

In some instances the Fretlight® showed an advantage during training. That is, the Fretlight® seemed to provide a lower barrier of entry for learning the guitar as supported by higher note quality during the first training trial, lower amounts of wrong notes played during earlier training trials, lower inconsistency in time between notes, and lower total scale times during earlier trials. However, by the end of training, participants performed equally well across the training trials regardless of condition. Taken together, these results lend partial support to H1. However, the effects were not quite as clear during the testing phase of the experiment.

With regard to testing, when the Fretlights® or diagram was removed, the pattern of results differed from those found in training. Specifically, results for note quality during Test Trial 1 showed that scale note quality in the Fretlight® condition was initially lower than the diagram condition, though both followed the same pattern of change over time. However, the results also showed an interaction between condition and time practicing the scale. At the midpoint of the test trials, the diagram condition was more prone to errors than the Fretlight®. On the one hand, findings suggest an initial benefit of traditional scale diagram (i.e., at Test Trial 1) with regard to note quality but that this benefit diminishes over time. On the other hand, the findings suggest a benefit for Fretlights® when considering errors during instances in which either learning method is unavailable. Therefore, the results of our test trials lend partial support to our H2 hypothesis. In short, the training data indicates that the Fretlight® may provide a lower barrier to entry for learning the guitar; however, both conditions ended up performing similarly on the test trials.

From a theoretical perspective, many of these findings can be discussed in light of our revised version of Leman's (2008) Action-Reaction Cycle (see Figure 2). As described, given that the Fretlight® provides music information on the neck of the guitar, the need to perceive and transform distributed learning materials is reduced. In other words, the findings of shorter total scale time and inconsistency between notes lend support to the idea that the Fretlight® guitar contributes to more of an embodied engagement in learning that is conceptually situated within the rightmost circle of the revised cycle. However, the results are less clear with regard to the effects of the overall cycle, and our proposed deviating loop through the sub-cycle. For example, if our interpretation is correct, than it would seem that the rightmost circle of the cycle is beneficial early on during learning, but, when the training scaffold is removed, going through the sub-cycle has some benefits (i.e., initial test performance was better with the diagram but then converged with Fretlight®). We elaborate more on these ideas in the general discussion.

## Experiment 2

Experiment 2, which was conducted parallel to Experiment 1, used a longitudinal design to examine how learning with the Fretlight® guitar moderates retention of previously learned scales across a longer time interval than Experiment 1 (i.e., 2 weeks). Similar to Experiment 1, participants learned the A minor pentatonic scale with either the Fretlight® or the traditional diagram method. Our hypotheses were as follows:

“H1: Participants will demonstrate long term learning effects across both conditions via the DVs, scale note quality, errors, inconsistency between notes, and total scale time.”

“H2: Participants who learn to play the A minor pentatonic scale with the aid of the Fretlight® will have significantly greater rates of improvement over time when compared to the diagram condition across scale note quality, errors, inconsistency between notes, and total scale time.”

### Participants

Six participants (2 female, 4 male; *M*_age_ = 29.83, *SD*_age_ = 5.85, age range 24–39) from a large mid-western university voluntarily participated in this study. To participate in this study individuals must have been right-handed and must have stated that they did not have any formal or informal guitar training.

### Materials

Similar to Experiment 1, a Black FG-521 Traditional Electric Guitar was used for both conditions in the experiment. The program Fretlight® studio v5.02 was used to control the display of the LED lights for participants using the Fretlight® learning method. For those in the diagram condition, a paper diagram was provided to participants (see Figure 3). A Microsoft LifeCam Studio 1080 p HD was used to record both the video and audio of the training and test trials.

### Design

Experiment 2 utilized a within-subjects longitudinal design where six participants were trained and tested on retention in two measurement periods across 3-weeks. During Session 1, the experimental design was akin to Experiment 1. Half of the participants learned the A minor pentatonic scale with the Fretlight® and half with the diagram. Participants completed 30 training trials using the Fretlight® or the diagram training. Participants were required to complete 10 test trials during Session 1, and then during Session 2 were tested on scale retention 2-weeks later. In addition, this experiment used similar dependent variables as Experiment 1 (*scale note quality, inconsistency between notes, and total scale time*). However, due to audio distortions on some of our participant's video files, *scale note quality* was replaced with *scale accuracy.* This was is operationalized as a correct note versus incorrect note. Therefore, every training/test trial received a score of 0–12 (i.e., 1 point per correct note of the 12 note A minor pentatonic in 5th position).

### Procedure

Experimental procedures during Session 1 were identical to Experiment 1 with the exception of the follow up session. Instead, after 2-weeks, participants came back to the laboratory for an additional A minor pentatonic testing session, where they were required to play 10 test trials without the aid of any learning method.

### Performance rating

Videos were analyzed by two undergraduate research assistant raters. The assistants rated each trial using the quality of note scale implemented in Experiment 1. As described above, due to audio distortions, the assistants were asked to rate each scale with the correct note versus incorrect note method where every training/test trial received a score of 0–12, with 1 point possible for each of the notes in the 5th position A minor pentatonic scale. Due to a smaller sample size, assistants rated every training and test trial. As in Experiment 1, *ICC* was used to assess the reliability across the two assistants. Once all trials were rated for all participants, inter-rater reliability was assessed as excellent, with an *ICC* = 0.90.

### Experiment 2 training performance results

#### Scale accuracy

**Figure 6:**
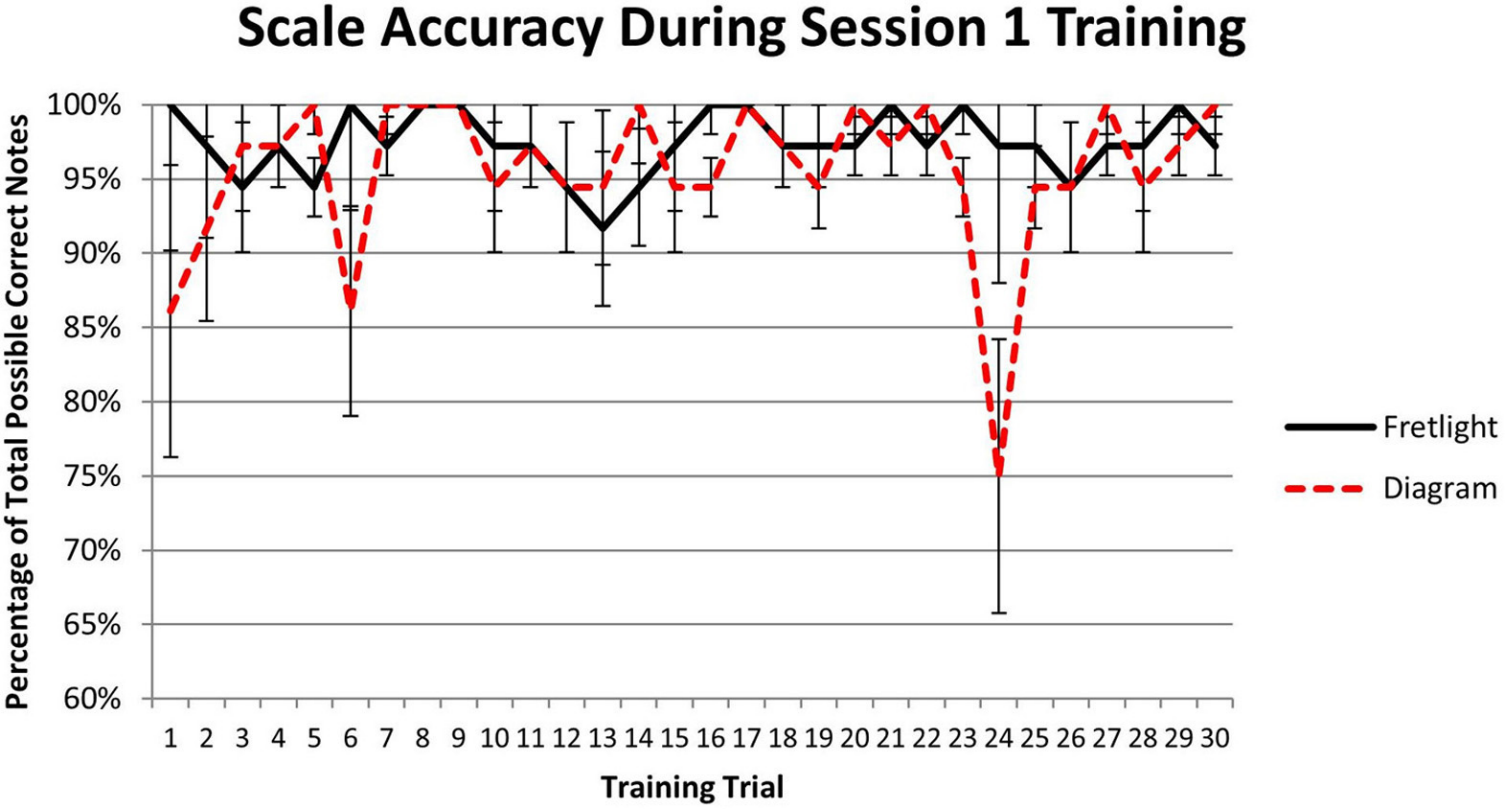
**Experiment 2 results regarding scale accuracy during session 1 training**.

To compare training performance across the learning methods, we used a 2 × 30 mixed model ANOVA. There were no effects for scale accuracy, including both main effects [training, *F*_(29,116)_ = 0.98, *p* = 0.51, η^2^_*p*_ = 0.196; learning method, *F*_(1, 4)_ = 1.56, *p* = 0.279, η^2^_*p*_ = 0.281]. Further, the interaction effect was also non-significant: *F*_(29, 116)_ = 0.22, *p* > 0.05, η^2^_*p*_ = 0.216 (Figure 6). Therefore, scale accuracy did not vary by learning method or as participants practiced the scale across time.

#### Inconsistency between notes

**Figure 7:**
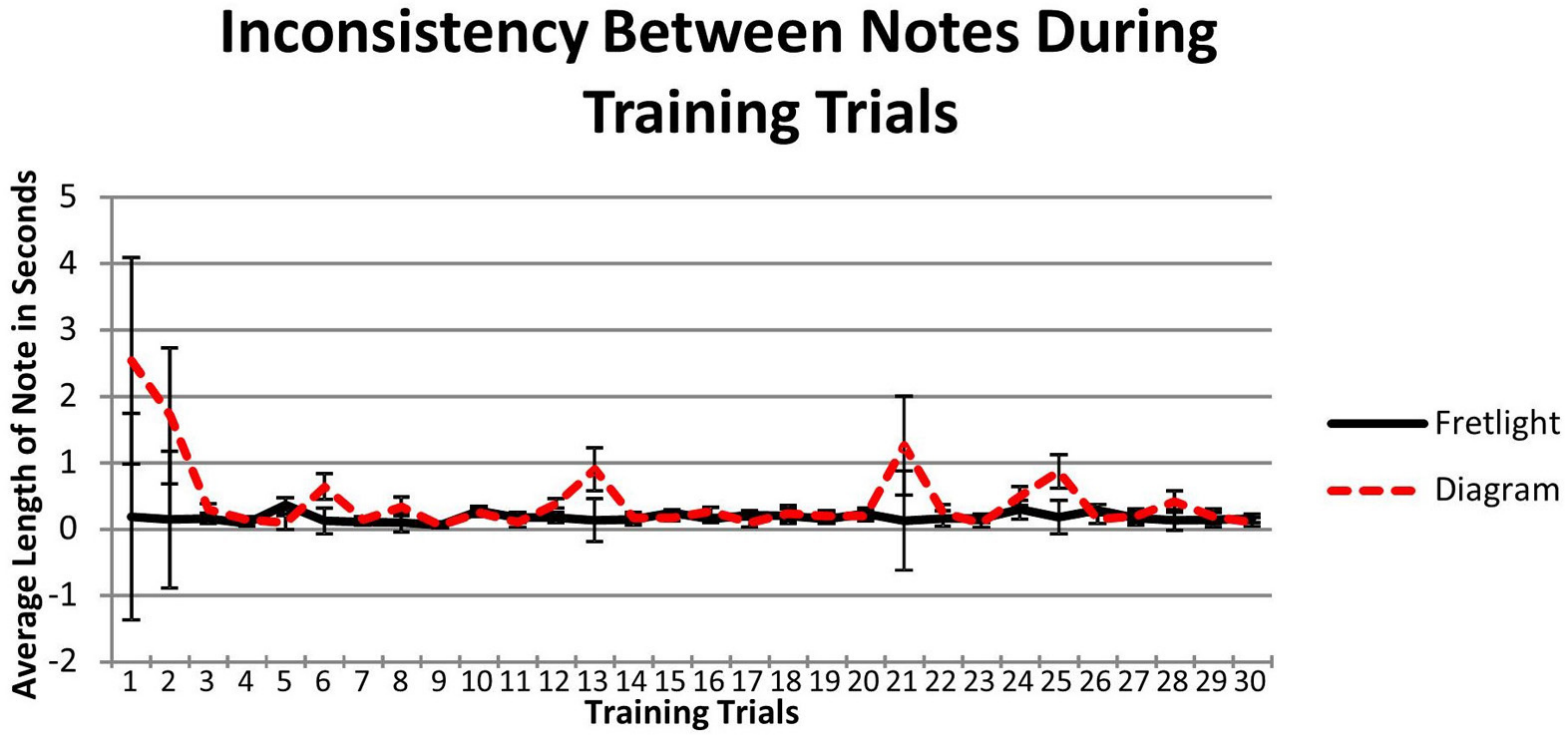
**Inconsistency between notes during training trials for Experiment 2**.

Inconsistency between notes during training was analyzed using a 2 × 30 mixed model ANOVA. Again no effects were significant. Specifically, there was no main effect across training trials, *F*_(29, 116)_ = 1.05, *p* > 0.05, η^2^_*p*_ = 0.21, nor a main effect between groups, *F*_(1, 4)_ = 3.04, *p* = 0.172, η^2^_*p*_ = 0.408. Furthermore, there was no observable interaction between training and learning method, *F*_(29, 116)_ = 1.10, *p* > 0.05, η^2^_*p*_ = 0.22 (Figure 7). This means that inconsistency between notes did not vary across training trials or learning methods.

#### Total scale time

**Figure 8:**
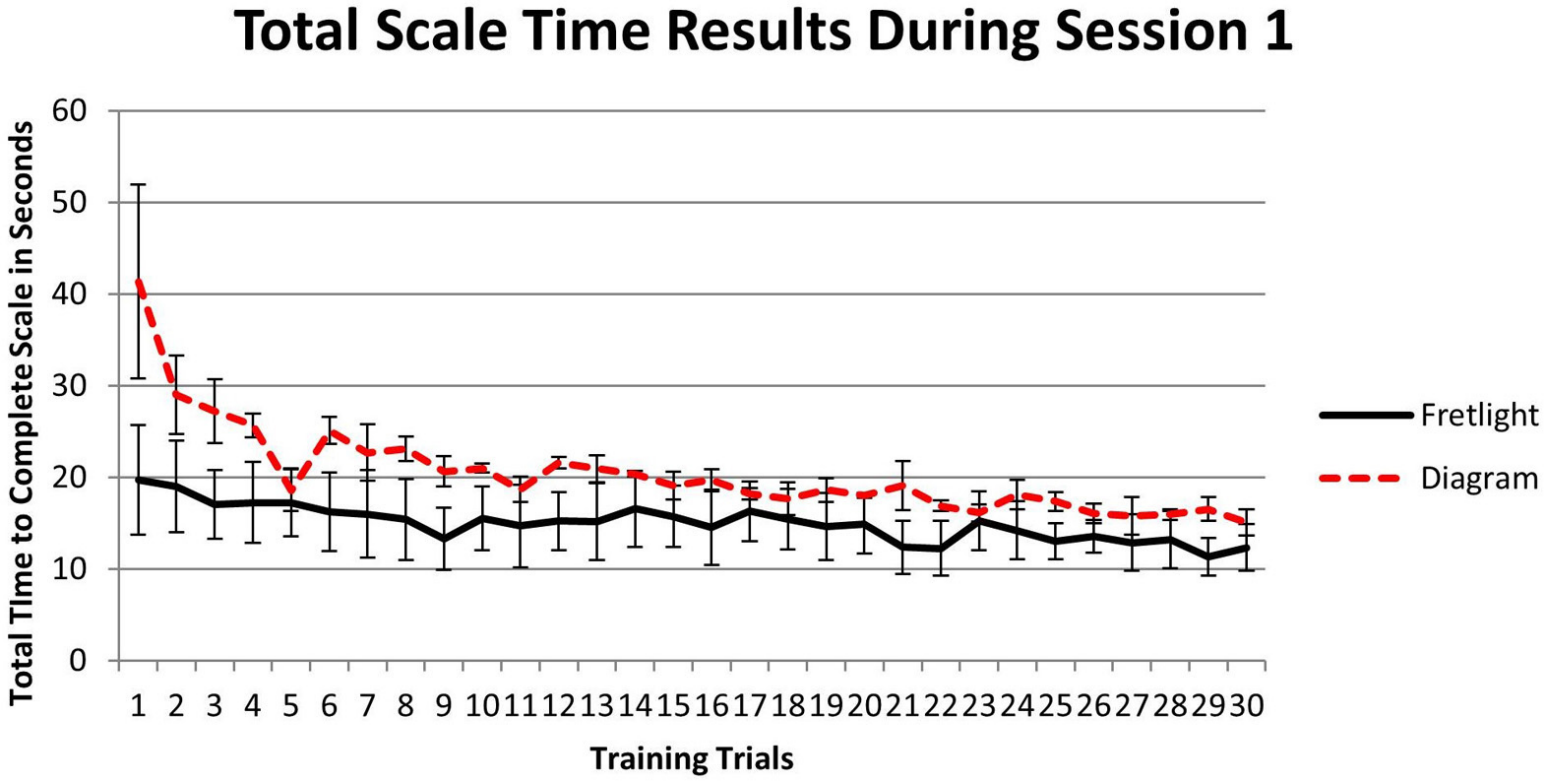
**Time to complete each training trial by learning method**.

To examine the relationship between learning method and total scale time during training, we utilized a 2 × 30 mixed model ANOVA. Results indicated a significant main effect for total scale time across training trials, *F*_(29, 116)_ = 5.78, *p* < 0.001, η^2^_*p*_ = 0.591. That is, participants played the scale in less time as they progressed through the training trials. However, there was not a main effect between groups, *F*_(1, 4)_ = 2.44, *p* = 0.193, η^*2*^_p_ = 0.379. There was an interaction effect, *F*_(29, 116)_ = 1.88, *p* = 0.01, η^2^_*p*_ = 0.32, such that participants who used the Fretlight® had accelerated performance, and could play the scale more quickly as they progressed through the trials when compared to the diagram condition (Figure 8). Last, test for simple effects showed that training Trial 29 was significantly different between learning conditions, *p* < 0.05.

### Experiment 2 test performance and scale retention results

#### Scale retention

**Figure 9:**
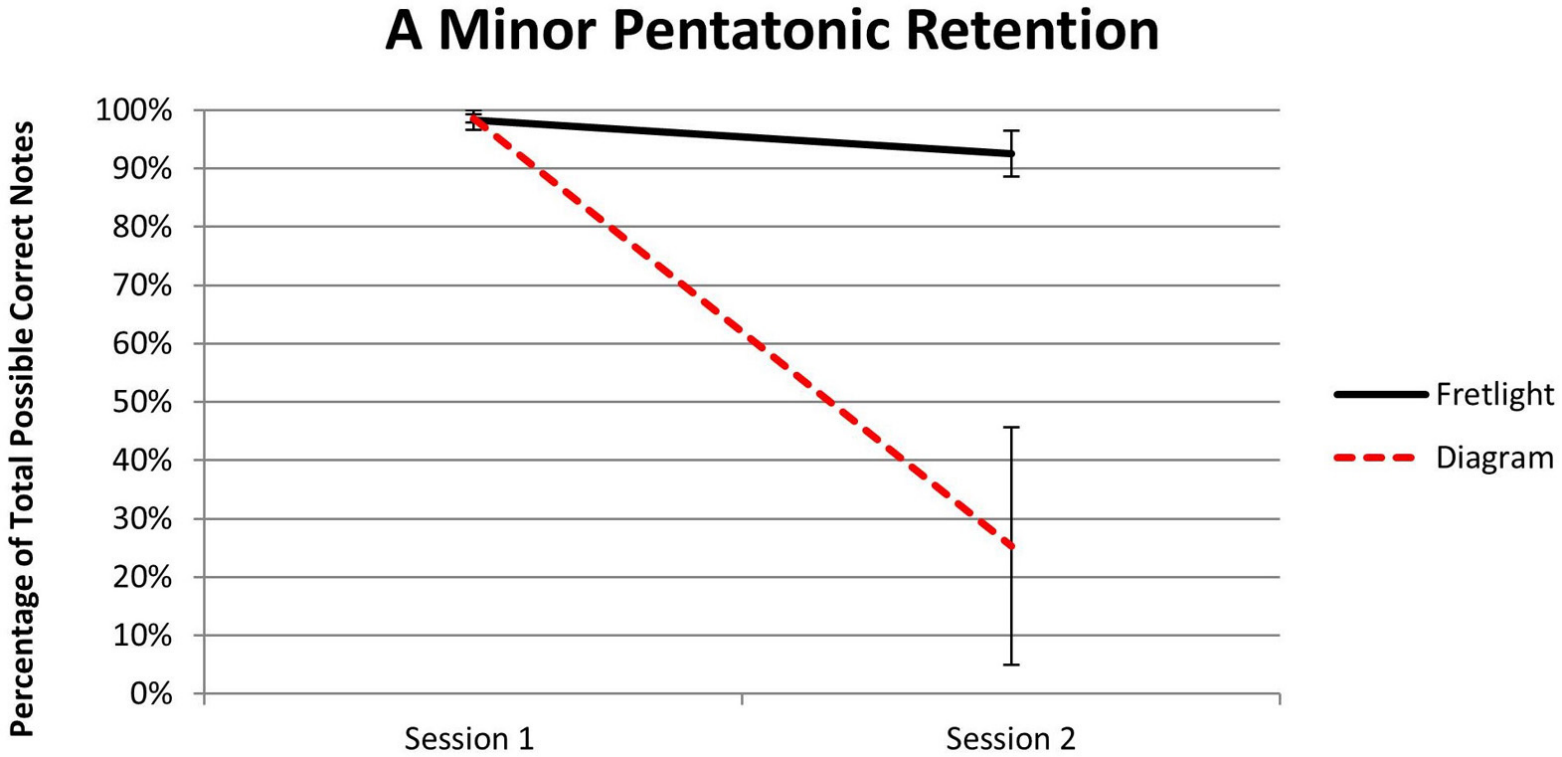
**Experiment 2 results regarding A minor pentatonic scale retention**.

A 2 × 2 mixed model ANOVA comparing the learning effects of the two conditions across two test measurement periods revealed that, overall, participants trained using the Fretlight® guitar performed significantly better on the test trials across both sessions, *F*_(1,4)_ = 14.45, *p* = 0.02, η^2^_*p*_ = 0.78. In addition, there was a main effect between groups, *F*_(1, 4)_ = 10.34, *p* = 0.03, η^2^_*p*_ = 0.72. This suggests that participants in the Fretlight® condition remembered significantly more notes of the A minor pentatonic compared to the diagram group. Results also indicated a significant interaction effect, *F*_(1, 4)_ = 10.50, *p* = 0.03, η^2^_*p*_ = 0.72. A test for simple effects showed participants trained using the Fretlight® had significantly higher retention of the A minor pentatonic scale during the second test measurement period when compared to the rest of the sample, *t*_(4)_ = 3.24, *p* < 0.02, η^2^_*p*_ = 0.72 (Figure 9).

#### Inconsistency between notes

**Figure 10:**
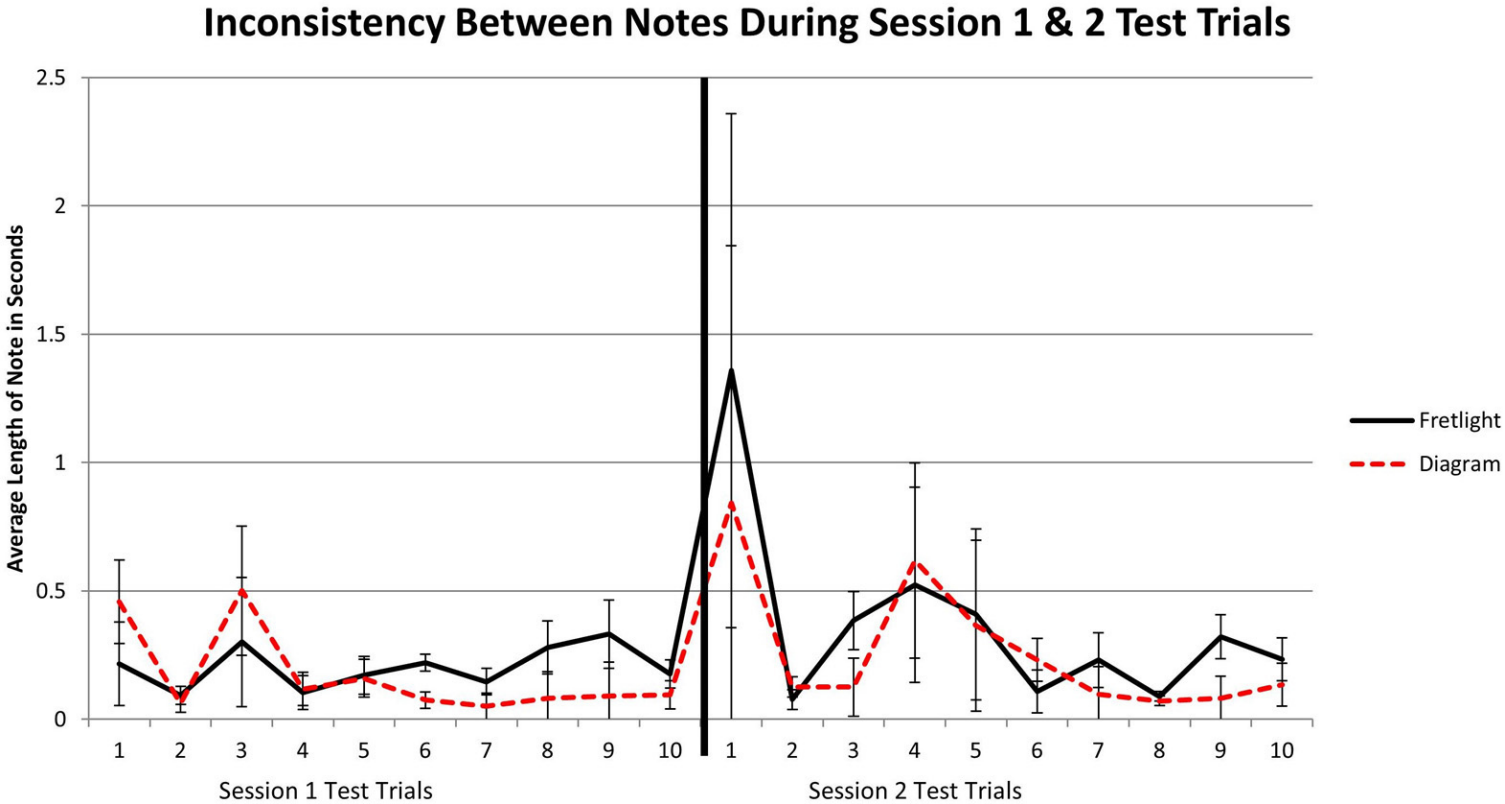
**Inconsistency between notes during test trials for Experiment 2 across session 1 and 2**.

Inconsistency between notes during the two test trials was analyzed using a 2 × 20 mixed model ANOVA. There was not a significant main effect for test trials, *F*_(19, 76)_ = 1.58, *p* = 0.08, η^2^_*p*_ = 0.283. In addition, there was not a main effect between groups, *F*_(1, 4)_ = 0.45, *p* = 0.53, η^2^_*p*_ = 0.104. Finally there was no interaction effect, *F*_(19, 76)_ = 0.22, *p* > 0.05, η^2^_*p*_ = 0.05 (Figure 9). There were no differences between the two conditions, nor across testing trials, for inconsistency between notes (Figure 10).

#### Total scale time during session 1 testing

**Figure 11:**
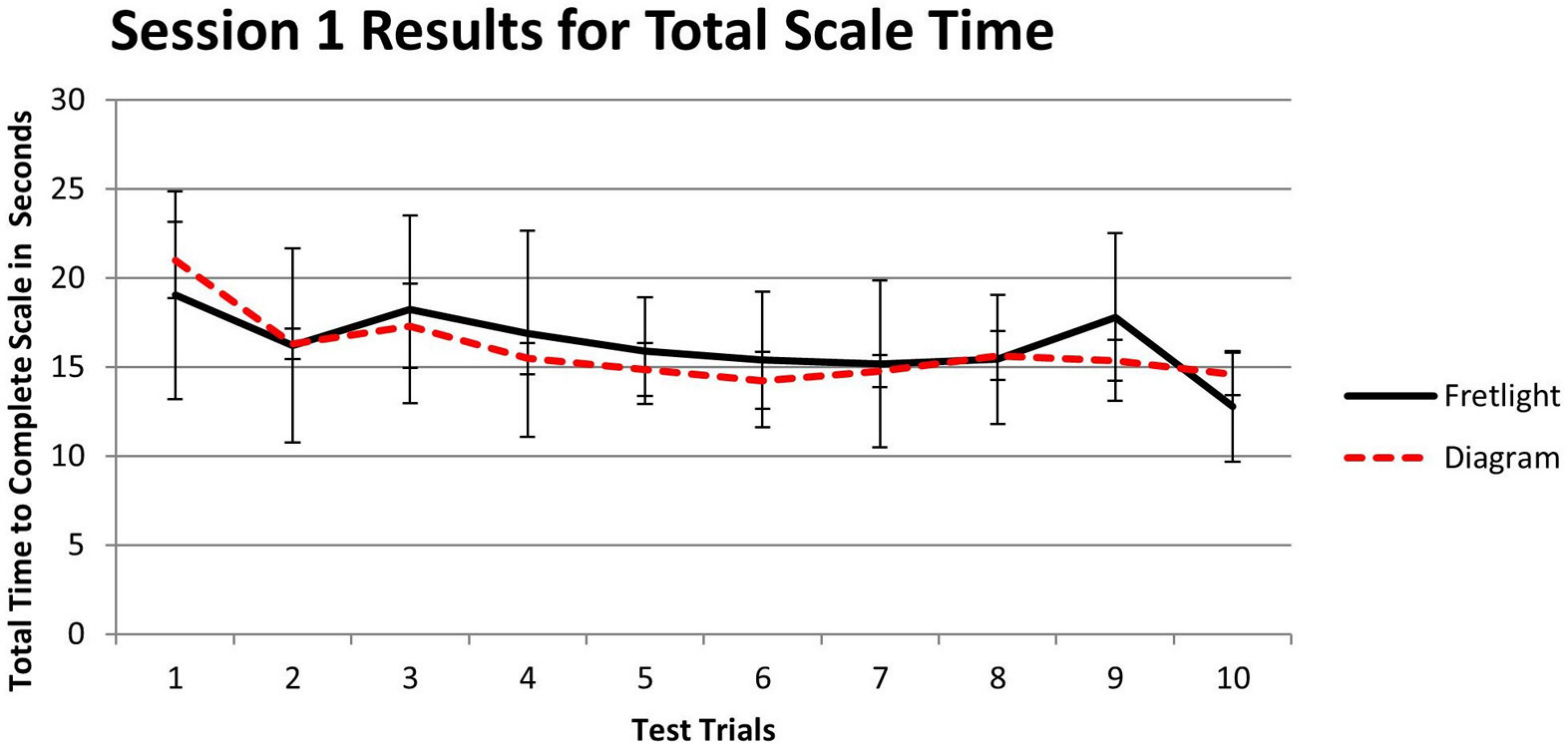
**Time to complete each immediate test trial by learning method**.

To examine the relationship between learning method and total scale time during the Session 1 testing period (i.e., immediately after training), we utilized a 2 × 10 mixed model ANOVA. We observed a significant main effect for trial time, *F*_(9, 36)_ = 3.91, *p* = 0.002, η^2^_*p*_ = 0.494. Participants played the scale in a shorter amount of time as they progressed through the testing trials. However, there was no main effect between groups, *F*_(1, 4)_ = 0.01, *p* = 0.95, η^2^_*p*_ = 0.001, and no observable interaction effect between trial time and learning method, *F*_(9, 36)_ = 0.61, *p* > 0.05, η^2^_*p*_ = 0.133 (Figure 11). Therefore, the different learning methods did not appear to affect the total scale time during the first testing period.

#### Total scale time during session 2 testing

**Figure 12:**
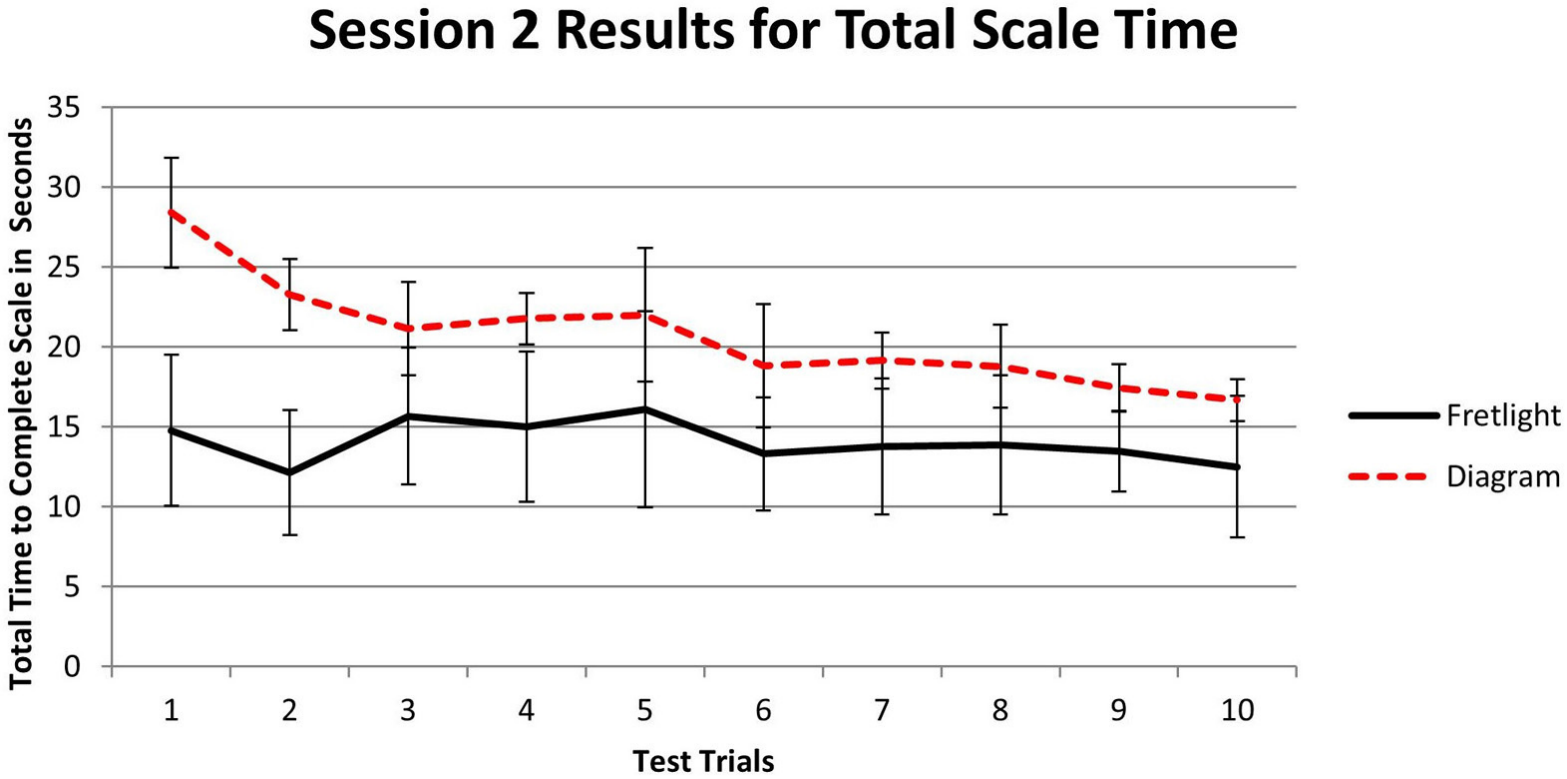
**Time to complete each test trial by learning method after 2-weeks**.

To examine the relationship between learning method and total scale time during Session 2, we utilized a 2 × 10 mixed model ANOVA. A significant main effect for trial time was found, *F*_(9, 36)_ = 3.99, *p* = 0.002, η^2^_*p*_ = 0.491. Participants played the scale in a shorter amount of time as they progressed through the trials. However, there was no main effect between groups, *F*_(1, 4)_ = 1.97, *p* = 0.23, η^2^_*p*_ = 0.33. The training conditions did not differ during the second testing period. In addition, there was an interaction effect for time to complete a trial, *F*_(9, 36)_ = 0.2.29, *p* = 0.04, η^2^_*p*_ = 0.364. Participants who utilized the Fretlight® during scale training accelerated to faster scale times during testing when compared to those who learned using the diagram (Figure 12). After a test for simple effects we identified retention Trials 1 and 2 as significantly different across learning method conditions; *t*_(4)_ = 2.34, *p* < 0.05; *t*_(4)_ = 2.48, *p* < 0.05.

### Experiment 2 discussion

Experiment 2 demonstrated support for both of our hypotheses although in some instances the effects were not clear (Table 2). There were longitudinal learning effects for both groups, as predicted by H1. Specifically, both groups had higher performance across time as indicated by the results of inconsistency between notes and total scale time during training. The effect on total scale time between notes indicated a significant interaction between trial and the Fretlight® condition. Consistent with Experiment 1, this demonstrates that although the Fretlight® may provide a lower barrier of entry, the diagram condition appears to “catch up” by the end of the training we provided. Therefore, to better understand the effects of the system requires the examination of effects over longer durations, such as after the 2-week interval.

**Table 2:** **Summary of Experiment 2 Results**.

**DV**	**Effect**
Scale accuracy	Training trials
	Learning method
	Training trials X learning method
Scale retention	Time^*^
	Learning method^*^^FL^
	Time X learning method^*^^FL^
Training inconsistency between notes	Training trials
	Learning method
	Training trials X learning method
Testing inconsistency between notes	Time
	Learning method
	Time X learning method
Training total scale time	Training trials^*^
	Learning method
	Training trials X learning method^*^^FL^
Session one testing total scale time	Test trials^*^
	Learning method
	Test trials X learning method
Session two testing total scale time	Test trials^*^
	Learning method
	Test trials X learning method^*^^FL^

* *p < 0.05*,

Interaction effects are qualified with superscript to indicate which condition showed an advantage where ^FL^, Fretlight®, ^D^, Diagram.

The findings from longitudinal measurement (i.e., 2-weeks post training) further demonstrate the beneficial effects of the Fretlight® learning system compared to the diagram. Specifically, those in the Fretlight® condition had significantly better performance outcomes as predicted by H2. The effects found for scale accuracy and total scale time during Session 2 indicate that the Fretlight® condition had lasting learning performance effects when compared to the diagram condition across the 2-week interval.

As with the discussion for Experiment 1, we believe many of the H1 effects can be theoretically characterized by the revised Leman's (2008) Action-Reaction Cycle. As noted in the discussion of Experiment 1, learning with the Fretlight® seems to necessitate more of an embodied engagement as illustrated with the rightmost loop of the cycle (Figure 2). Again, Experiment 2 lends credence to the reduced need to perceive and transform distributed music materials as evidenced by shorter total scale time during training with the Fretlight®.

The results of Experiment 2 indicate that, although all participants initially performed at approximately the same level, the 2-week time span led to decrements in performance for the diagram group. In contrast, those in the Fretlight® group maintained the same level of performance as they had during the first experimental session. These findings provide preliminary evidence that the Fretlight® may provide both a lower barrier of entry as well as improved long-term retention, both of which are indicators of learning benefits over normal instruments. From a theoretical perspective, these findings support the idea that a stronger embodied engagement, with less need to engage in mental transformation characterized by the proposed sub-cycle (Figure 2), may lead to better learning effects (cf. Johnson-Glenberg et al., 2014). Below we discuss both experiments in the context of our overarching research program and theoretical insights. We also discuss limitations of the present work and ideas for future research.

## General discussion

Across two experiments, we found a number of effects contrasting two distinct learning methods. Consistent with general principles of learning, we find that regardless of condition, all participants' performance increased across time spent practicing the scale. But, the Fretlight® system appears to provide a lower barrier of entry to playing the guitar as evidenced by findings where performance was better for the Fretlight® during earlier training trials. Further, use of the Fretlight® led to shorter total scale times during training and testing. In addition, while the diagram may have had slightly better test performance than the Fretlight® system initially, by the end of the test performances the two conditions were approximately equivalent. Further, when trained with the Fretlight® system, participants appeared to have greater long-term retention of the scale.

Broadly, the evidence provided by these two experiments demonstrates that, when the musical material to be learned becomes embodied and embedded within the instrument itself this may lower the barriers of entry for learning and enhance long-term retention of the learned musical information. Specifically, when contrasted to a standard musical diagram for learning a guitar scale, the Fretlight® system had somewhat consistent effects across a number of dependent variables characterizing participant learning performance. Although this research is in the preliminary stages, we find this evidence to be promising.

These results have novel theoretical implications for embodied music cognition in the context of learning an instrument that embeds the required spatial-temporal information for the learner. These experiments provide evidence supporting the notion that such technologies can meaningfully advance the state of the art in musical instrument learning. Given these technologies will only continue to advance, research is warranted to practically inform the design of such novel music instrument learning technologies, and to contribute to evolving theorizing on embodied music cognition.

In our introduction, we discussed varying perspectives of embodiment and how they might relate to musical instrument learning (e.g., grounded cognition and the ecological perspective). While we did not test the predictions that each account might make, we hope that our inclusion of them here might make such research more accessible. In addition, the theoretical and empirical research space encompassed by embodied music cognition is vast. With our work we have attempted to specify a niche within this space that can be characterized as *embodied music technology for learning*.

Within this niche, our major contribution to the theoretical discussion is the revised version of Leman's (2008) Action-Reaction Cycle model (see Figure 2). In short, we have updated the model to account for music instrument learning specifically when it involves examination of learning material distributed in the environment vice embedded within the instrument. By no means do we feel our revision to the model is conclusive, and we thus encourage others to critique and refine our ideas. Indeed, we proposed this model as a theoretical scaffold for our empirical work. Of course, further work in this vein, whether theoretical or empirical, may show that the reality is much more complex (cf., Sterman, 2002). Drawing from discussion of Leman's (2008) original model, we recognize that the Action-Reaction cycle is a dynamically occurring and emergent process characteristic of playing a music instrument in which “a set of action-reaction cycles may occur at different time scales and perhaps in hierarchical order” (p. 54). Although Leman made this statement in the context of developing a musical instrument, the same likely holds true in terms of the cycles occurring at different time scales and hierarchies characterizing the process of learning to play an instrument.

To reiterate, the additional sub-cycle we added to the Action-Reaction Cycle characterizes the ways that an individual perceives external learning materials, describes the ways in which they transform them from the external representation onto their instrument, and then engage in a verification of the accuracy of the transformation (see Figure 3). The distinction of import here is that traditional external learning materials necessitate cognitive transformations where abstraction notational diagrams need to be translated into psycho-motor behavior and executed on the instrument (cf. Norton et al., 2005). This is in contrast to our point about *embodied music technology for learning*, such as the Fretlight® guitar. Here, although still an external learning material, this should reduce the need for transformations because the information is provided on the instrument itself. Importantly, this leads to an important theoretical and empirical question as to whether or not there is a deeper level of processing associated with the initiation of this transformational sub-cycle (cf., Craik and Lockhart, 1972). Indeed, our results lend partial credence to this idea as it may help to explain why learning with the diagram was beneficial at the initial test trial (Experiment 1).

To further elaborate on this idea, recall we discussed that learning techniques that rely on internalization are initially more difficult, but may lead to better long-term retention; whereas, externalized learning techniques may be less beneficial in the long term, but provide a lower level of initial difficulty (Van Nimwegen et al., 2004). It may be that learning with the diagram provides a higher degree of internalization when compared to the Fretlight® guitar. This was reflected by the early performance difference favoring the Fretlight® condition. Some evidence for why this might be the case can be found in recent work on mental practice. Here, merely engaging in mental practice (e.g., movement accuracy and velocity for experienced piano players performing a complex musical piece) was facilitative (Bernardi et al., 2013a). Nonetheless, performance was still best when physically practicing the piece. Although it is difficult to disentangle mental and physical practice from our experiments, the additional transformation process required to memorize the diagrams, could provide mental practice when compared to those in the Fretlight® condition. This could be assessed in future research by comparing the responses on the dimensions of the mental strategies questionnaire (Bernardi et al., 2009, 2013a,b) between the two learning conditions.

But our long-term retention findings differ from those of Van Nimwegen et al. (2004). If the diagram condition, as we posited above, necessitates a greater degree of internalization, then it would have shown better performance on the 2-week retention test (Experiment 2). This, however, was not the case. So perhaps it is the case that the Fretlight®, in providing for a stronger coupling between what to play, where to play it, and what it should sound like, may lead to better retention than the higher degree of explicit memorization that could be associated with the diagram. Such speculations provide additional avenues for further research.

In sum, through lowering the barrier of entry, increasing retention of musical information, and potentially making the instrument easier to learn, the Fretlight® guitar or similar augmented instruments could improve informal musical learning. Additional support for this claim comes in part from the recent efforts of McDermott et al. (2013) who note specifically that the methods used for music instruction should be made easier with a particular emphasis on more personalized and intelligent computer systems that help to refine and enhance the types of practice musicians employ, as well as serving as a foundational means for long-term engagement with the instrument. The Fretlight® guitar may be particularly useful to individuals who may have given up at early phases of instrument learning. If using the instrument studied here, they may be able to reach new levels of learning and performance. And beginners, who would normally be discouraged, might learn more rapidly and be motivated to continue practicing with the instrument. Overall, then, the system mitigates the need to learn complex music notation systems that require a high-degree of internalization and, more often than not, the assistance of a teacher at earlier stages. Of course, we in no way support the idea that a musical instrument should be learned absent from pedagogy, teachers, or some form of music notation system. But a system like the Fretlight® could reinforce such instruction or inspire individuals to continue playing to the point where they will actively seek a teacher or develop the capacity to learn more traditional forms of music notation once they have reached a certain level of competence.

### Limitations

The two experiments discussed provide preliminary evidence demonstrating the utility of an augmented reality system for musical learning, something we have coined as an *embodied music technology for learning*. These experiments are not without limitation in their methodologies. Specifically, both experiments relied only on the A minor pentatonic scale. The simplicity of this six note scale may have led to a ceiling effect, where all participants performed well during training regardless of condition.

Further, Experiment 2 had a relatively small sample size (*N* = 6). Although there were significant differences, it may be argued that this sample is not representative of the population. Future research, discussed in more detail below, will need to examine results with a larger sample to conclude if these effects indeed exist.

A potential confound to consider for future research is musical background. Although we required that participants not have any stringed instrument training, we did fully control for musical background. So some participants with musical experience, outside the realm of stringed instruments, may have affected our results. One way to address this would be to utilize an auditory skills test (see Bernardi et al., 2013a,b; for an example). Participants could also be asked to detail their experience with any instrument or music theory and this could be covaried into any analyses. Another potentially informative way to address this might be to use the Music Experience Questionnaire (Werner et al., 2006) as this could help to address general individual differences with regard to participant interactions with music. More generally, future experiments could examine how demographic data (e.g., age, gender, handedness) along with individidual differences (e.g., spatial aptitudes, music interests, etc.) and assess how these may moderate results. Finally, the environment and style of learning and performing studied in these experiments were highly controlled, and do not necessarily represent the way individuals learn or perform, formally or informally, in the real world. This will need to be mitigated both during training and testing in future studies.

### Future research

Although preliminary, the evidence provided in this manuscript is promising. Yet, there is still much to be done to ensure that the learning effects found here are valid. In light of the Action-Reaction Cycle as described by Leman (2008), we believe that our updated model (Figure 2) is a closer approximation of processes involved in musical learning. This will need to be tested further.

As discussed in the limitations section, some of the null effects found in Experiment 1 could very well be due to the ease of playing the five note A minor pentatonic scale. To aid in clarifying these effects, future studies should vary complexity of learning material (e.g., 7 note scales and song riffs) to better understand the differences between learning materials when the content is difficult. Along these lines, it would also be interesting to compare participant's ratings of the difficulty of learning with the various instructional methods as an index of how participants assess their own learning processes. The application of cognitive load theory (Paas et al., 2003) could be used as a basis for this type of research.

Also, future research needs to understand the differential effects of learning on these systems when the participants are at different levels of expertise. For instance, the system could lead to a decrement in performance or handicap an expert who has achieved fluency with more standard methods, such as reading guitar tablature or music notation. It would be interesting to better understand exactly how an expert guitarist would use the system, if at all. Further, having experts learn novel material with the system could provide insight into the way expert mental models differ from novices, and this could aid in bridging the gap to expertise through the development of better technological training tools and practices.

Additionally, the software associated with the Fretlight® system provides a rich set of musical tools that are ripe for the types of user-oriented studies with both experts and novices that Leman and colleagues propose for further investigating embodied music cognition (e.g., Leman et al., 2010).

Further, future research will need to better understand the effects of this type of device in more formalized learning settings. The studies here provided a learning experience that was under the need of experimental control, leading to learning processes that were informal and in some ways unrealistic. Future research will need to aim at creating more realistic applied environments for understanding the way individuals learn during formalized lessons and how they perform under more realistic constraints.

In light of needing to better understand training, future research should also focus on scaffolding approaches using such systems. For instance, how does one learn using the Fretlight®, and then transfer this knowledge to performance? What methods are best to ensure long-term retention and high levels of performance in the absence of the embodied information? Perhaps, it is the case that there are inherent benefits to both novel and traditional instrument learning techniques and the proper sequence of theses may have the best learning outcome. In this respect, although the Fretlight® does provide direct information on the neck, it does not provide a structured training program. It may be up to either the system designers or music teachers to implement best practices for scaffolding training. This could range, for example, from learning scales and songs with the Fretlight® system turned on, to a student becoming competent enough in the absence of the information to play the instrument in a performance. Another question that also remains to be answered with regard to novel methods for music instrument training is if the generalized cognitive benefits that stem from music training (e.g., Aagten-Murphy et al., 2014; see Miendlarzewska and Trost, 2014 for review) would still remain with the use of these novel technologies.

In closing, as novel music instruction systems are developed, the tight coupling of theoretical discussion surrounding embodied music cognition and empirical evaluation of learning with such technologies, whether formal or informal, may just be the catalyst for a paradigmatic shift in music instruction.

### Conflict of interest statement

The authors declare that the research was conducted in the absence of any commercial or financial relationships that could be construed as a potential conflict of interest.

## Supplementary material

Supplementary Material. Performance Rating Scale Employed in Experiment 1
