# Supplementary material for: Shifting the paradigm of music instruction: implications of embodiment stemming from an augmented reality guitar learning system
Source: Front Psychol. 2014 May 27;5:471. doi: 10.3389/fpsyg.2014.00471 (PMC4034341; doi:10.3389/fpsyg.2014.00471)
Supplement: Supplementary file 1 [file DataSheet1.DOCX]

**Appendix A. Performance Rating Scale Employed in Experiment 1**

| **Quality of Correct Notes** | **Errors** |
| --- | --- |
| **4pt** = Correct note – Very clean sound  **3pt** = Correct note – Some unclean sound (slight buzz)  **2pt** = Correct note - Very unclean sound (lots of buzzing)  **1pt** = Correct note - No sound or highly muted  **0pt** = No note at all (skips note in scale) | - *Wrong note* - Plays a note that is not within the scale) - *Extra note* – Plays an extra note in addition to the 12 notes in scale, including repeated playing of a given note multiple times |
